# DAXX promotes ovarian cancer ascites cell proliferation and migration by activating the ERK signaling pathway

**DOI:** 10.1186/s13048-018-0462-4

**Published:** 2018-10-18

**Authors:** Sheng-Bing Liu, Xue-Ping Lin, Ying Xu, Zhong-Fei Shen, Wei-Wei Pan

**Affiliations:** College of Medicine, Jiaxing University, Jiaxing, 314001 China

**Keywords:** DAXX, Ascites cell, Cell proliferation, Cell migration

## Abstract

**Background:**

The death-domain-associated protein (DAXX) was originally identified as a protein that binds to the transmembrane death receptor FAS and enhances both FAS-induced and transforming growth factor-β-dependent apoptosis. In a previous study, we found that nude mice injected with DAXX-overexpressing cells (ES-2-DAXX) accumulated large concentrations of first-generation ascites cells (I ascites cells). The role of DAXX in the development of ascites is unknown. The aim of this study was to analyze the effect of DAXX on proliferation and migration of ascites cells in ovarian cancer in vitro and in vivo.

**Methods:**

Nude mice were housed in cages with a 14:10 h light:dark cycle; water and food were provided ad libitum. ES-2-DAXX cells (1×106) were injected intraperitoneally into athymic nude mice (8-week-old female mice). After 4 weeks, I ascites cells were collected. The I ascites cells were injected intraperitoneally into athymic nude mice (8-week-old female mice). After 4 weeks, II ascites cells were collected and cultured. Ascites cell survival, migration, and colony formation were measured using colony formation and cell growth assays. Immunofluorescent staining revealed the co-localization of DAXX and promyelocytic leukemia protein (PML) in ascites cell nuclei. Western blotting and immunohistochemistry showed that extracellular signal-related kinase (p-ERK) 1/2 and CEBP-β were highly expressed in tumor tissues formed by II ascites cells. Through immunoprecipitation, we also found that DAXX can interact with CEBP-β.

**Results:**

DAXX enhanced ascites cell survival, migration, and colony formation. DAXX and PML nuclear foci dramatically increased in a passage-dependent manner in ascites cells, DAXX promoted the tumor growth of ascites cells in vivo, increased ascites cell proliferation in vivo, and enhanced ascites cell survival and migration by activating the ERK signalling pathway and integrating with CEBP-β.

**Conclusions:**

DAXX can interact with CEBP-β. DAXX can induce ovarian cancer ascites formation by activating the ERK signal pathway and binding to CEBP-β.

## Introduction

Ovarian cancer is the fifth most common cause of death in women, and the second most common gynecological cancer worldwide [1]. Serous ovarian cancer is the most common subtype; most patients present with late-stage tumor and dissemination of tumor implants throughout the peritoneal cavity [2]. Ascites creates a protective environment for ovarian tumor cells that inhibit drug-induced apoptosis [3, 4]. Ascites is described as heterogeneous fluids that display marked differences in their levels of soluble factors; some of these factors can potentially activate an array of signaling pathways [5].

Ascites formation is an important problem in patients with ovarian cancer. The development of ascites is a hallmark symptom of late-stage ovarian cancer, and biologically active factors within the fluid can affect disease progression and interfere with anti-tumor vaccination strategies [6, 7]. The mechanism of ovarian cancer ascites formation is unknown. In a previous study, we found that DAXX-overexpressing cells (ES-2-DAXX) accumulated large concentrations of ascites cells [8]. Based on these data, we found that DAXX-overexpression-induced ascites cells had an increased colony number and migration ability in vitro. We further demonstrated that the ERK pathway was activated in DAXX-overexpression-induced ascites cells. We also found that DAXX interacted with CEBP-β in DAXX-overexpression-induced ascites cells. Our results indicated that DAXX promotes ovarian cancer ascites cell proliferation by activating the ERK pathway and directly binding to CEBP-β.

## Methods

### Reagents and cell culture

ES-2 cells (human ovarian cancer cell lines) purchased from the ATCC (Manassas, VA, USA) were cultured in DMEM (Dulbecco’s Modified Eagle’s Medium) which contains 10% fetal bovine serum and 1% penicillin-streptomycin solution (Hyclone, GE Healthcare, Little Chalfont, UK) at 37 °C in a humidified 5% CO_2_ incubator.

### Plasmids and Daxx-overexpressing ovarian cancer cell lines

The plasmid of pLEGFP-*Daxx* which encode the full-length *Daxx* sequence and HA-CEBP-β were kindly provided by Zhejiang University Professor Fan Heng-yu. Overexpressed ES-2-DAXX cells were established using the methods described previously [8].

### Ascites cells and xenograft models

Nude mice were housed in cages with a 14:10 h light:dark cycle; water and food were provided ad libitum*.* The NIH Guides for the Care and Use of Laboratory Animals were used as all animal protocols. ES-2-DAXX (The abbreviation of ES-2-DAXX cells is ES-DAXX in all figures.) (1 × 10^6^) were injected intraperitoneally into athymic nude mice (8-week-old female mice), ovarian cancer ascites cells in vivo were obtained through the above experiments.After 4 weeks, ascites cells were collected and centrifuged at 157 g for 10 min. The acellular fractions were cultured in DMEM which contained 10% fetal bovine serum mediums and 1% penicillin-streptomycin in a humidified 5% CO_2_ incubator at 37 °C. These cells were designated I ascites cells.The I ascites cells (1 × 10^6^) were injected intraperitoneally into athymic nude mice (8-week-old female mice). After 4 weeks, II ascites cells were collected and cultured. Nude mice were injected with the BrdU solution to a concentration of 100 mg/kg. Two hours later, primary tumor, ovaries, and intestinal masses were collected from athymic nude mice, these primary tumor and organs were fixed with 4% formalin and made into slices (5-μm thick), then the slices were stained with HE (hematoxylin and eosin) staining.

### Colony formation assay

Single ascites cell suspensions were prepared and seeded. Colonies were counted after 10 days in 60-mm dishes. The dishes were cultured in triplicate in a 5% CO_2_ humidified incubator at 37 °C.

### Transwell migration assay

For the transwell migration assay, 24-well plate inserts with 8-μm pore filters, the migration was assessed with BioCoat Matrigl (BD Biosciences, San Jose, CA, USA). 5 × 10^4^cells was added to serum-free medium and suspended in a transwell. Ascites cells stably transfected with DAXX at the upper surface of the transwells were removed after cells were incubated for 24 h at 37 °C. Migrated cells were stained with H&E staining and rinsed with double distilled water, then the transwells were air-dried. The positive cells were counted by ImagePro Plus 6.0 software.

### Cell growth assay

Cell proliferation was assessed by MTT assay. Ascites cells were cultured and seeded in plates, the cell densities of the 96-well plates was 3 × 10^3^ cells/well. After the cell adhered to the wall, the 96-well plates was added 20 μl /well MTT solution (5 mg/ml in PBS) and was put into incubator in 37 °C for 4 h. Absorbance value was tested using micro-plate reader at 490 nm.

### Soft agar colony formation assay

Cells were cultured using the method of Hamburger and Salmon with modifications. 1.5 ml of 0.5% agar were prepared in 6-well. Cells were suspended in 1.5 ml of 0.35% agar containing 1 × cell culture medium and 10% fetal bovine serum and poured over these underlayers. The final cell concentration in each culture was 0.5 × 10^3^ cells/ml. Triplicate cultures were used for each experiment. The plates were placed in a 5% CO_2_ humidified incubator at 37 °C. Colonies were counted at 2 to 3 weeks after plating using an Omnicon FAS II Image Analysis System.

### Immunohistochemistry

Paraffin-embedded human ovarian tumor tissues were obtained from the Jiaxing Maternity and Child Health Care Hospital, China. The archived human ovarian tumor tissues used in the experiments had the Jiaxing University Institutional Review Board approval. 5-μm sections were made, referring to ABC kit (Vector Laboratories, Burlingame, CA, USA), sections stained includes the following aspects: deparaffinized, rehydrated, and antigen retrieval, after the sections were incubated in H_2_O_2_ (0.3%) for 10 min, the sections were incubated in 10% goat serum for 30 min. Then the sections stained with affinity-purified anti-BrdU, anti-DAXX (Sigma Aldrich, St. Louis, MO, USA), anti-p-AKT, anti-p-ERK (Cell Signaling Technology, Danvers, MA, USA), and anti-CEBP-β (Santa Cruz Biotechnology, Dallas, TX, USA) antibodies (1:200 dilution), The antibodies were incubated for 1 h. After washing with PBS, the sections were incubated with a secondary antibody for 30 min and washed again with PBS before incubation with avidin-biotin complex (ABC) solution. The sections were stained through DAB (diaminobenzidine) (DAB substrate kit, Vector Laboratories) staining.

### Immunofluorescence

Nude mouse tumor tissues were gained to make microtome section, the tissues embedded in OCT compound (Sakura Finetek USA, Torrance, CA, USA) after fixed in 4% paraformaldehyde, then Leica CM1850 cryomicrotome (Leica Microsystems) was used to slice. Ascites cells of nude mouse were washed with PBS after the cells were seeded on coverslips for 24 h, then ascites cells were fixed and permeabilized, 5% BSA was used as blocking reagent. The ascites cells were incubated with primary antibodies at room temperature for 1 h. The sections of ascites cells were stained through DAPI.

### RNA extraction and real-time RT–PCR analysis

Total RNA was extracted from cultured cells using Trizol reagent (Invitrogene), according to the manufacturer’s instructions. Real-time PCR analysis was performed using TB Green Premix qPCR kit (TaKaRa, Japan) and an Applied 7300 Real-Time PCR System. Relative mRNA levels were determined by normalizing to the endogenous *Gapdh* mRNA.

- *Gapdh*:5′-gcctggagaaacctgccaagtatg-3′; 5′-gagtgggagttgctgttgaagtcg-3′;
- *CEBP-β*: 5′-gacaagcacagcgacgagta-3′; 5′-agctgctccaccttcttctg-3’
- *Fog2*: 5′-tggggacacacagtcagaga-3′; 5′-cctcagagatggccttcgta-3′
- *Tff1*: 5′-gaaggtcatgtcaagggaggt-3′; 5′-atgagcttgcaccacgttct-3’

### Western blotting

The proteins extracted from ascites cells were separated by SDS-PAGE and transferred to PVDF membranes, then the membranes were incubated with 5% BSA for 1 h at room temperature. The membranes were incubated with primary antibodies for 1 h at room temperature and the horseradish peroxidase-conjugated goat anti - rabbit antibodies were used as second antibodies (Cell Signaling Technology). After incubated for 1 h at room temperature, the membranes were washed with TBST. Bound antibodies were visualized through ECL Western Blotting chemiluminescence reagent kit (Amersham, GE Healthcare).The primary antibodies dilution was1:1000, antibodies which were tested included DAXX (Sigma Aldrich), ERK, CEBP-β, p-ERK and β-actin (Cell Signaling Technology).

### Immunoprecipitation

To detect protein-protein interactions, the vector HA-CEBP-βwere transfected into ascites cells. After transfection, Ascites cells were lysed 48 h using NP-40 buffer. The lysates were centrifuged at 13,000×*g* for 25 min. By using anti-HA M2 affinity beads (Sigma Aldrich) for 4 h at 4 °C, immunoprecipitation was performed. The NTEN buffer was boiled in loading buffer (4 × SDS) after the beads were washed in NTEN buffer, then separated by electrophoresis (SDS-PAGE). The membranes were incubated with antibodies included DAXX, CEBP-β, and β-actin after incubated with 5% BSA.

### Statistical analysis

The in vitro assays results were repeated for three times. The statistical techniques employed for analysis are one-way ANOVA using GraphPad Prism statistical software (GraphPad Prism, La Jolla, CA, USA) and two-tailed T-tests. To consider *p* < 0.05 as statistically significant.

## Results

### DAXX enhances ascites cell survive, migration, and colony formation

**Fig. 1:**
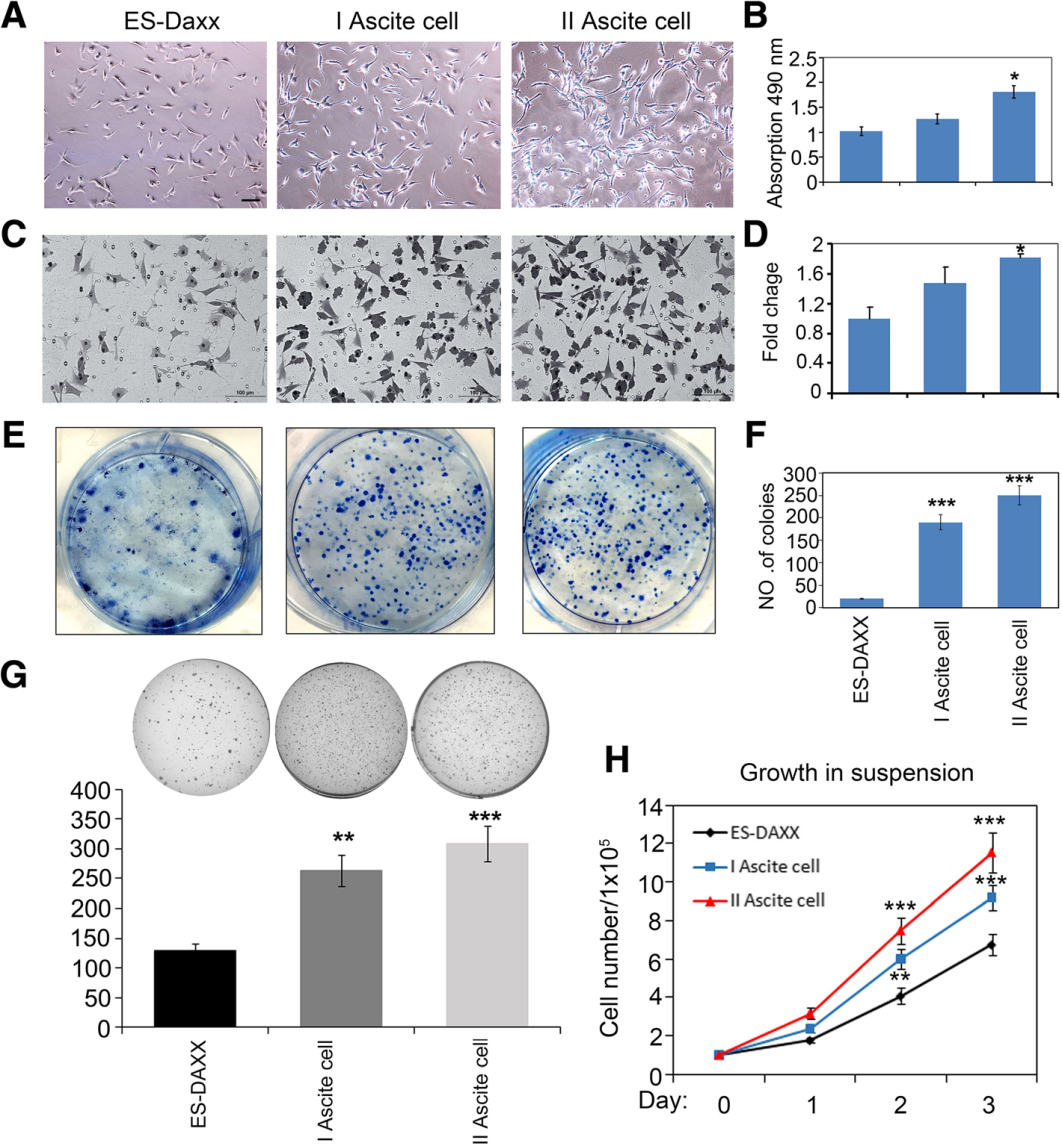
DAXX enhances ascites cell colony formation and migration. **a–b** DAXX-induced ascites cell proliferation. Ascites cells were seeded into 96-well plates (3,000 cells/well) overnight, and then assessed by an MTT assay. Scale bar, 50 μm. **c**–**d** Transwell experiment to determine the migration capability of ascites cells and their derivatives (GFP-DAXX-overexpressing ascites cells). Cells were added to transwells and allowed to migrate for 12 h. Cells at the upper surface of the membrane were removed with cotton swabs, and cells on the bottom surface were stained with hematoxylin and eosin. Scale bar, 100 μm. **e**–**f** Colony formation assay for the growth of ascites cells with DAXX overexpression. Colony numbers were counted after 10–14 days. * *p* < 0.05; ***, *p* < 0.0001. **g** DAXX overexpression promotes anchorage-independent growth of ascite cells in vitro. Soft-agar colony-formation assay was performed and the colonies were stained with crystal violet for quantification. **h** DAXX overexpression promoted cell proliferation in suspension (detachment) conditions. Cells (1 × 10^5^) were plated in 6-well culture dishes and cell number was determined with trypan blue staining. Three replicates were included. The error bars represent s.d. ns (*p* > 0.05); *** *p* < 0.001. Student’s t-test was applied

To investigate the role of DAXX in ascites cells, we developed first- and second-generation ovarian cancer ascites cells (I ascites cells and II ascites cells) with stable green fluorescent protein (GFP)-DAXX expression. First, we investigated the role of DAXX in ascites cell survive, migration, and colony formation. As shown in Fig. 1a–b, II ascites cells grew faster than the parental GFP-DAXX overexpression cells, as assessed by MTT assays. The role of DAXX on ascites cell migration was also determined. We found that overexpressed DAXX markedly promoted ascites cells migration (Fig. 1c–d). Furthermore, DAXX expression in ascites cells strongly promoted colony formation (Fig. 1e–f). Notably, one important criterion distinguishes malignant cancer cells from “normal” cells is their ability to grow in soft agar. 3D cell cultures are well documented to better mimic the in vivo situation than monolayer cell culture on plastic [9–11]. Then, we compared the cell growth of ES-2-DAXX cells, I ascites cells and II ascites cells under soft agar and anchorage-independent conditions (in cell suspension). We found that II ascites cells showed a significantly stronger growth potential than ES-2-DAXX cells in soft agar and suspension (Fig. 1g–h). These results demonstrate that DAXX promoted ascites cell proliferation and migration in a passage-dependent manner.

### DAXX and promyelocytic leukemia protein (PML) nuclear foci dramatically increase in a passage-dependent manner in ascites cells

**Fig. 2:**
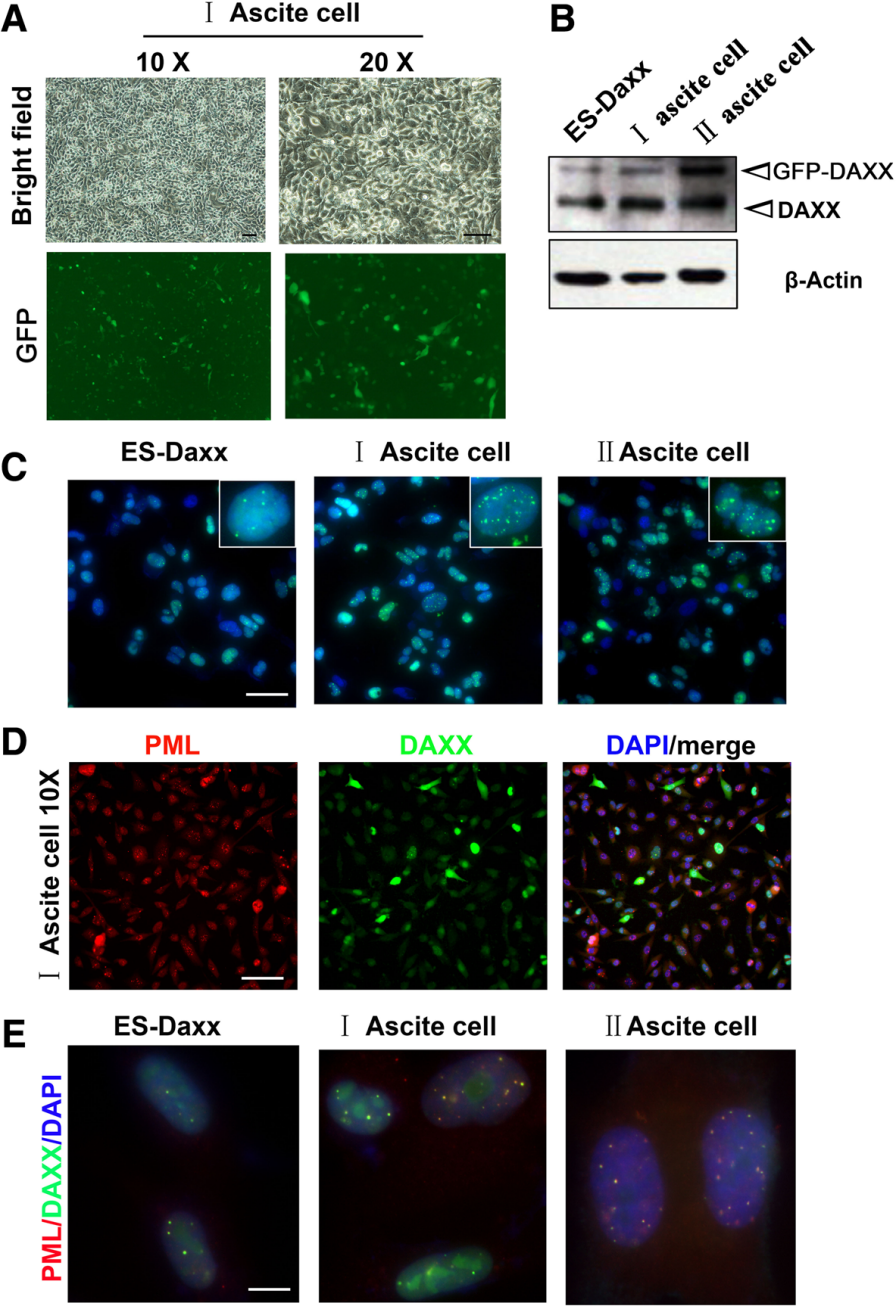
DAXX and PML nuclear localization in ascites cells. **a** Ascites cell morphology using brightfield and GFP microscopy (10 × and 20× magnification, respectively). **b** Western blot results for DAXX overexpression in ascites cells. **c** Ascites cells were harvested from nude mice and cultured in vitro. Fluorescence microscopy showing the localization of overexpressed DAXX (green) in ascites cells stably transfected with GFP-DAXX. Nuclei were stained with 4′,6-diamidino-2-phenylindole (DAPI; blue). *Scale bar*, 50 μm. **d** Immunofluorescence results for promyelocytic leukemia protein (PML) foci formation in ascites cells. *Scale bar*, 50 μm. **e** Immunofluorescence results for PML, DAXX and DAPI in ES-Daxx, I and II ascites cells. *Scale bar*, 10 μm

To explore the role of DAXX on ascites cells, we harvested and cultured ascites cells in DMEM medium (Fig. 2a). Western blot examination revealed that the ascites cells were positive for GFP-DAXX (Fig. 2b), which illustrated that ascites cells were derived from the parental ES-Daxx cells. Through immunostaining, we found that DAXX nuclear aggregation significantly increased in a passage-dependent manner (Fig. 2c). In our previous study, we found that DAXX co-localized with PML in ovarian cancer cells and mouse ovarian surface epithelium (mOSE) [12]. Here, we sought to quantify PML expression in ascites cells through immunofluorescence staining. We found that PML exhibited nuclear location in ascites cells; this distribution was consistent with DAXX in ascites cells nuclei (Fig. 2d–e).

### DAXX promotes the tumor growth of ascites cells in vivo

**Fig. 3:**
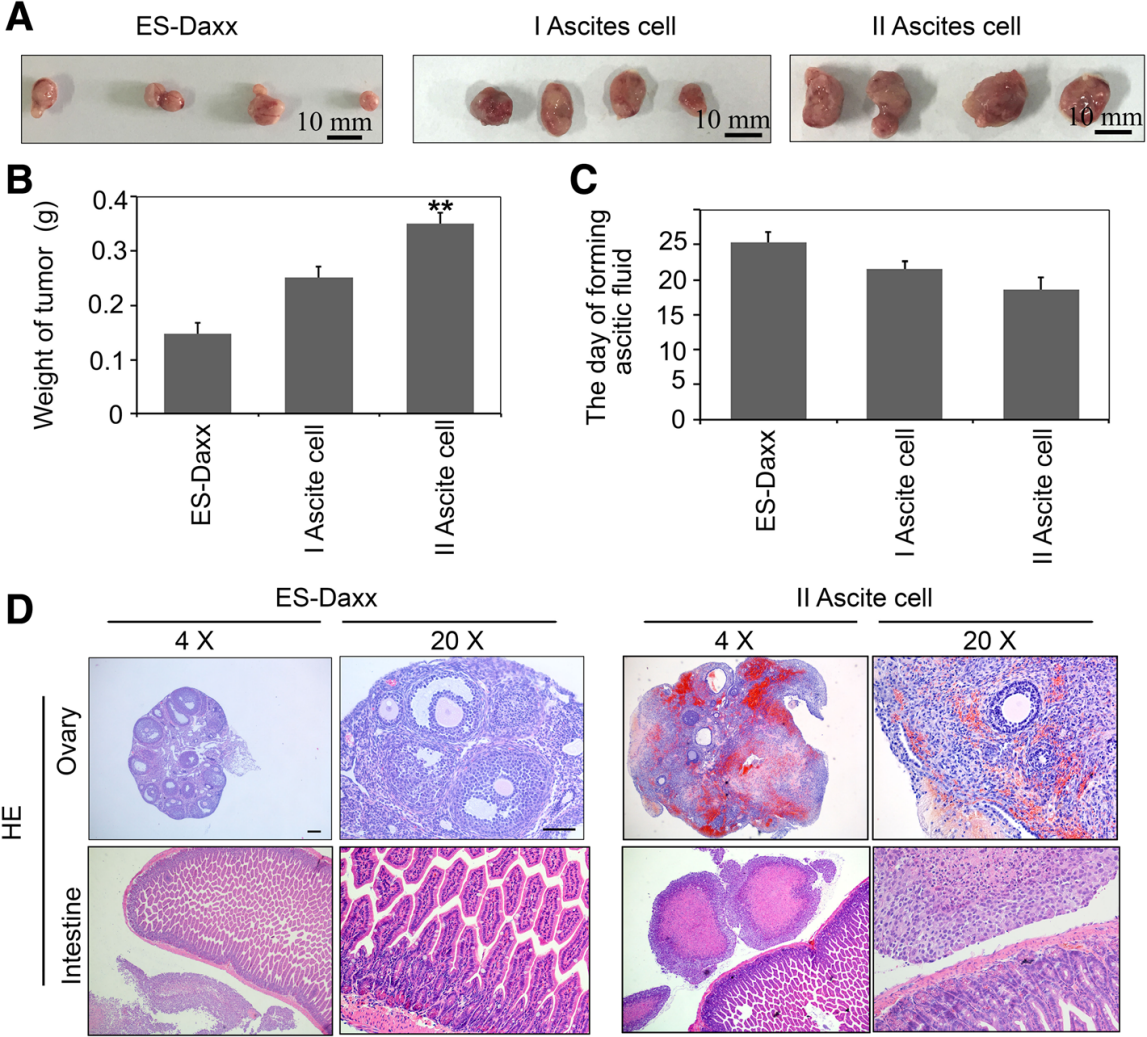
DAXX promotes ascites cell metastasis in vivo. **a–b** Ovarian cancer cells (ES-Daxx) and I and II ascites cells (10^6^cells for each) were implanted intraperitoneally into different nude mice. After 20 days, tumors were removed and weighed (*n* = 5). **c** Quantify ascites formation time (d). **d** ES-Daxx and II ascites cells were injected intraperitoneally into nude mice (10^6^ cells/mouse). HE staining showing cells metastasis to the intestine and ovary 3 weeks later. *Scale bar*, 250 μm

We also detected the role of DAXX in promoting the tumorigenic and metastatic capabilities of ascites cells. Female nude mice were injected intraperitoneally with either parental ES-2-DAXX cells or ascites cells. As shown in Fig. 3a–b, the average weight of the abdominal solid tumors formed by II ascites cells was approximately threefold greater than the average weight of the control formed by the parental ES-2-DAXX cells. The ascites formation time decreased in a passage-dependent manner (Fig. 3c). Examination of the peritoneal cavities of mouse that injected II ascites cells showed a significant and dispersed distribution of tumor exposites in the ovary and intestine (Fig. 3d).

### DAXX increases ascites cell proliferation in vivo

**Fig. 4:**
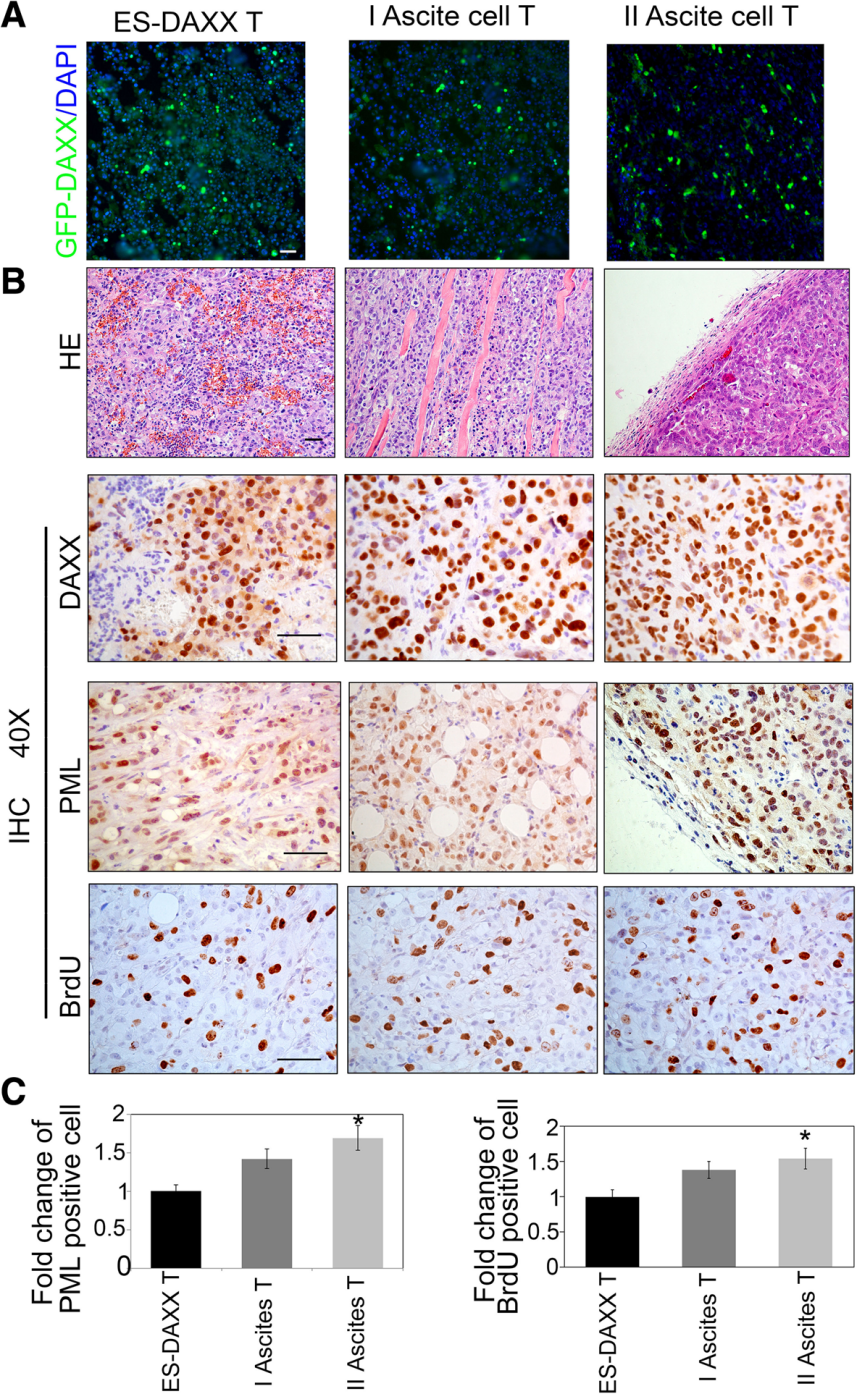
DAXX promotes ascites cell proliferation in vivo. **a** Cryosections were prepared from tumor tissues derived from GFP-DAXX-overexpressing ascites cells. **b** HE and Immunohistochemistry staining for DAXX, PML, and BrdU in representative DAXX-overexpressingascites cells. *Scale bar*, 50 μm. **c** Quantify PML and BrdU positive cells in B. *, *p* < 0.05

To illuminate the role of DAXX on ascites cells proliferation, we observed GFP expression. Fluorescence microscopy showed that the most of the tumor cells were derived from ES-2-DAXX ascites cells (Fig. 4a). Immunohistochemical examinations showed that DAXX and PML were all significantly expressed in ascites cell-derived tumors (Fig. 4b). Next, we measured bromodeoxyuridine (BrdU) levels through immunohistochemical staining. We found that BrdU was present in high concentrations in ascites cell-derived tumors (Fig. 4c). These results showed that DAXX promotes ascites cell proliferation in vivo.

### DAXX enhances ascites cell survival and migration by activating the ERK signaling pathway and integrating with CEBP-β

**Fig. 5:**
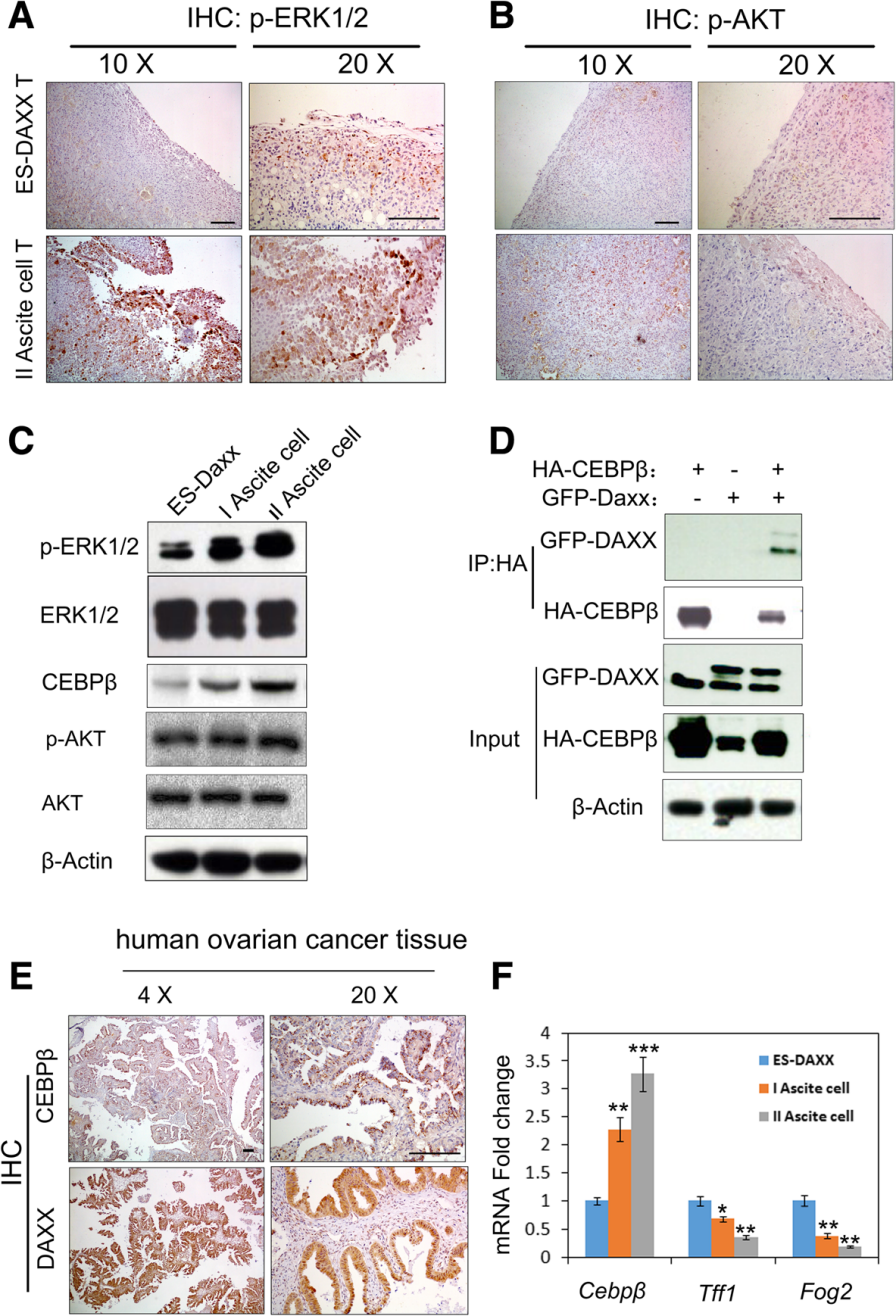
DAXX binds to CEBP-β and promotes ascites cell proliferation in vivo. **a–b** Immunohistochemistry staining for p-ERK1/2 and p-AKT in mouse tumor tissue. *Scale bar*, 200 μm. **c** DAXX-overexpressing ascites cells were subjected to immunoblotting analysis with antibodies against the indicated proteins. **d** DAXX-overexpressing ascites cells were transfected with the indicated combinations of plasmids encoding HA-CEBP-β. Cells were lysed after 24 h, and protein extracts were immunoprecipitated with anti-HA antibodies, followed by SDS-PAGE and western blotting. **e** Immunohistochemical analysis of DAXX and CEBP-β levels in human ovarian tumor tissue samples (*n* = 10). Sections were counterstained with hematoxylin and eosin. *Scale bar*, 200 μm. **f** *Cebpβ*, *Tff1* and *p16* mRNA expressions in ES-Daxx, I and II ascites cells were determined by Q-PCR

To understand how DAXX expression promoted ascites cells growth, we measured p-ERK1/2 and p-AKT expression in ascites cells tumors using immunohistochemistry. As shown in Fig. 5a–b, p-ERK1/2 was highly expressed in II ascites cell tumors. However, p-AKT was found to be minimally expressed in II ascites cell tumors. In order to confirm these results, we measured p-ERK1/2 expression in ascites cells using a western blot. We found that p-ERK1/2 and CEBP-β were highly expressed in a passage-dependent manner (Fig. 5c). A previous study showed that CEBP-β directly interacts with DAXX [13]. Next, we investigated the interaction between DAXX and CEBP-β by co-immunoprecipitation in ascites cells. As shown in Fig. 5d, DAXX was found to be co-immunoprecipitated with CEBP-β. After confirming that DAXX interacts with CEBP-β in ovarian cancer ascites cells, we investigated the expression of CEBP-β in human ovarian cancer tissues by immunohistochemistry (Fig. 5e). We found that CEBP-β and DAXX were significantly expressed in human ovarian cancer tissues (Fig. 5e). To confirm the function of CEBP-β, we detect CEBP-β target gene TFF1 and Fog2 expression in ES-2-DAXX cells, I ascites cells and II ascites cells. We found that II ascites cells decrease TFF1 and Fog2 expression (new Fig. 5f). The results showed that CEBP-β transcriptional activity were increased in a passage-dependent manner. These results suggest that DAXX integrated with CEBP-β and promoted ascites cells survival and migration.

## Discussion

Ovarian cancer is the most lethal tumor of all human gynecological malignancies. It is estimated that 220,000 new cases of ovarian cancer and 140,000 deaths are reported every year [14]. Due to the lack of symptoms and adequate screening methods in early stages, more than 60% of patients are diagnosed with advanced stages of ovarian cancer [15]. Ovarian cancer has a specific transmission pattern that is different from other malignancies. Tumors generally diffuse in abdominal cavity; even after recurrence, it is mostly confined to the peritoneal cavity, where it can carry out immune-suppressive activity [16]. Furthermore, for effective immune therapy, identification of the tumor antigen is required; for cellular immune therapy, the epitope recognized by T-cells must be identified.

DAXX can be either a pro- or anti-apoptotic factor, depending on the cell type [17]. DAXX was initially identified as a cytoplasmic molecule that links Fas signaling to the c-Jun N-terminal kinase (JNK) pathway via apoptosis signal-regulating kinase 1 (ASK1) [18]. In addition to its role in the cytoplasm, DAXX acts as a transcriptional co-repressor in the nuclear compartment. DAXX has been found to be associated with multiple proteins involved in transcriptional repression, such as HDAC1, DNA methyltransferase 1 (DMNT1), and α-thalassaemia/mental retardation syndrome X-linked (ATRX) [19–21]. DAXX also suppresses the activity of several transcription factors, including ETS1, Pax3, glucocorticoid receptor (GR), and p53 [22–25]. DAXX-dependent recruitment of H3.3 into PML-NBs both in proliferating cells, thus establishing PML-NBs as important assembly points for newly synthesized H3.3 histones and regulated gene expression. H3.3 is found enriched at active genes, centromeric heterochromatin, and telomeres, and has been proposed to act as important carrier of epigenetic information [26]. In our previous study, we found that DAXX was significantly expressed in human ovarian cancer tissue and weakly expressed in normal ovarian tissue. Female athymic nude mice were injected intraperitoneally with ES-2-DAXX cells or DAXX silencing cell (ES-2 shDAXX). Nude mice that injected ES-DAXX cells accumulated large amounts of ascites. These results showed that DAXX-overexpression played important role in ovarian cancer ascites forming [8]. In this study, we found that ES-2-DAXX cells promoted ascites cells proliferation and migration. DAXX co-localized with PML in ovarian cancer cells nuclei. DAXX and PML nuclear foci dramatically increase in a passage-dependent manner in ascites cells. This result showed that the co-localization of DAXX and PML maybe increased ascites cell-related gene expression by epigenetic regulation and promoted ascites forming.

It is well known that ascites-induced signaling events trigger the activation of both the ERK1/2 and AKT pathways. MAPK pathways is present in all cancer cells and regulates cell differentiation, proliferation, migration, apoptosis, and inflammation [27, 28]. In mammals, the activation of the MAPK/ERK pathway requires Ras, a single G protein recruited by receptor and non-receptor tyrosine kinases [29]. Activation of the ERK1/2 pathway is also involved in tumor cell survival by coupling survival stimuli to transcription factors controlling gene expression. For example, higher levels of phospho-ERK1/2 in ovarian cancer cells were found to be associated with an increased resistance to cisplatin [30]. Our results also showed that DAXX induced ovarian cancer ascites cell proliferation through ERK activation. These results were consistent with a previous study [31].

Phosphatidylinositol-4,5-bisphosphate 3 kinase (PI3K)/AKT may be a key pathway in the regulation of cell survival under anchorage-independent stress [32]. Previous studies demonstrating AKT activation by ascites cells prompted us to investigate whether AKT was also involved in DAXX-overexpression-induced ascites. We measured the phosphorylation of AKT in tumor tissues by immunohistochemistry, and found that AKT was not activated in DAXX-overexpression-induced ascites cells. These results indicated that ERK1/2, but not AKT, plays a major role in DAXX-overexpression-induced ascites cell proliferation.

CEBP-β is involved in tissue-specific gene expression and participates in fundamental cellular processes such as proliferation and differentiation [33]. In addition, CEBP-β is capable of increasing the expression of several target genes including genes coding for cytokines such as IL-6, IL-4, IL-5 and TNFα and genes coding for drug resistance such as ABCC2 and ABCB1 [34, 35]. Our results clearly demonstrated that DAXX interacts with CEBP-β in ascites cells. This indicated that DAXX-CEBP-β promotes ascites cells growth by stimulating ascites formation, altering the components of the ascites or having any other chemokines inside the ascites that leads to such cell growth promotion. A further delineation of the mechanistic link between DAXX and CEBP-β should allow a better understanding of DAXX functions in ovarian cancer ascites cells.

## Conclusions

DAXX can induce ovarian cancer ascites formation by activating the ERK signal pathway and binding to CEBP-β.

## Abbreviations

ASK1: Apoptosis signal-regulating kinase 1
ATRX: ɑ-thalassaemia/mental retardation syndrome X-linked
CEBP-β: CCAAT/enhancer-binding protein-β
DAXX: Death-domain-associated protein
DMEM: Dulbecco’s Modified Eagle’s Medium
DNMT1: DNA methyltransferase 1
ERK: Extracellular signal-related kinase
GR: Glucocorticoid receptor
HDAC1: Histone deacetylase 1
MAPK: Mitogen-activated protein kinase
mOSE: Mouse ovarian surface epithelium
PML: Promyelocytic leukemia protein
